# Addressing the Third Delay in Saving Mothers, Giving Life Districts in Uganda and Zambia: Ensuring Adequate and Appropriate Facility-Based Maternal and Perinatal Health Care

**DOI:** 10.9745/GHSP-D-18-00272

**Published:** 2019-03-11

**Authors:** Diane Morof, Florina Serbanescu, Mary M. Goodwin, Davidson H. Hamer, Alice R. Asiimwe, Leoda Hamomba, Masuka Musumali, Susanna Binzen, Adeodata Kekitiinwa, Brenda Picho, Frank Kaharuza, Phoebe Monalisa Namukanja, Dan Murokora, Vincent Kamara, Michelle Dynes, Curtis Blanton, Agnes Nalutaaya, Fredrick Luwaga, Michelle M. Schmitz, Jonathan LaBrecque, Claudia Morrissey Conlon, Brian McCarthy, Charlan Kroelinger, Thomas Clark

**Affiliations:** a Division of Reproductive Health, U.S. Centers for Disease Control and Prevention, Atlanta, GA, USA.; b U.S. Public Health Service Commissioned Corps, Rockville, MD, USA.; c Department of Global Health, Boston University School of Public Health, Boston, MA, USA.; d Section of Infectious Diseases, Department of Medicine, Boston Medical Center, Boston, MA, USA.; e Baylor College of Medicine Children's Foundation-Uganda, Kampala, Uganda.; f Division of Global HIV and TB, U.S. Centers for Disease Control and Prevention, Lusaka, Zambia.; g Family Health Division, U.S. Agency for International Development, Lusaka, Zambia.; h Infectious Diseases Institute, College of Health Sciences, Makerere University, Kampala, Uganda.; i HIV Health Office, U.S. Agency for International Development, Kampala, Uganda.; j Division of Global HIV and TB, U.S. Centers for Disease Control and Prevention, Kampala, Uganda.; k Bureau for Global Health, U.S. Agency for International Development, Washington DC. Now with Boston Children's Hospital, Boston, MA, USA.; l Bureau for Global Health, U.S. Agency for International Development, Washington, DC, USA.; m Eck Institute for Global Health, University of Notre Dame, Notre Dame, IN, USA.

## Abstract

**Background:**

Saving Mothers, Giving Life (SMGL) is a 5-year initiative implemented in participating districts in Uganda and Zambia that aimed to reduce deaths related to pregnancy and childbirth by targeting the 3 delays to receiving appropriate care: seeking, reaching, and receiving. Approaches to addressing the third delay included adequate health facility infrastructure, specifically sufficient equipment and medications; trained providers to provide quality evidence-based care; support for referrals to higher-level care; and effective maternal and perinatal death surveillance and response.

**Methods:**

SMGL used a mixed-methods approach to describe intervention strategies, outcomes, and health impacts. Programmatic and monitoring and evaluation data—health facility assessments, facility and community surveillance, and population-based mortality studies—were used to document the effectiveness of intervention components.

**Results:**

During the SMGL initiative, the proportion of facilities providing emergency obstetric and newborn care (EmONC) increased from 10% to 25% in Uganda and from 6% to 12% in Zambia. Correspondingly, the delivery rate occurring in EmONC facilities increased from 28.2% to 41.0% in Uganda and from 26.0% to 29.1% in Zambia. Nearly all facilities had at least one trained provider on staff by the endline evaluation. Staffing increases allowed a higher proportion of health centers to provide care 24 hours a day/7 days a week by endline—from 74.6% to 82.9% in Uganda and from 64.8% to 95.5% in Zambia. During this period, referral communication improved from 93.3% to 99.0% in Uganda and from 44.6% to 100% in Zambia, and data systems to identify and analyze causes of maternal and perinatal deaths were established and strengthened.

**Conclusion:**

SMGL's approach was associated with improvements in facility infrastructure, equipment, medication, access to skilled staff, and referral mechanisms and led to declines in facility maternal and perinatal mortality rates. Further work is needed to sustain these gains and to eliminate preventable maternal and perinatal deaths.

## Abstract (precis)

Saving Mothers, Giving Life used 6 strategies to address the third delay—receiving adequate health care after reaching a facility—in maternal and newborn health care. The intervention approaches can be adapted in low-resource settings to improve facility-based care and reduce maternal and perinatal mortality.

## INTRODUCTION

Saving Mothers, Giving Life (SMGL) is a 5-year initiative designed to reduce deaths related to pregnancy and childbirth. SMGL used a coordinated approach targeting the 3 delays—seeking, reaching, and receiving adequate care—that contribute to maternal deaths.^1^ This article focuses on maternal and perinatal deaths due to the third delay, the lack of receipt of timely, adequate, and appropriate obstetric care at a health care facility (Figure).^2^ An estimated 75% of maternal deaths globally result from direct obstetric causes, with more than half attributed to hemorrhage, hypertensive disorders, and sepsis.^3^ Moreover, approximately 29% of newborn deaths in sub-Saharan Africa are attributed to intrapartum-related events.^4^ Facility-based maternal and newborn care, including access to skilled providers and neonatal resuscitation, improves the likelihood of maternal and infant survival.^5^^,^^6^ Although multiple socioeconomic and environmental factors affect maternal and neonatal survival, reducing the delay in receiving adequate and appropriate care at a health facility is key to improving health outcomes.^7^

**FIGURE:**
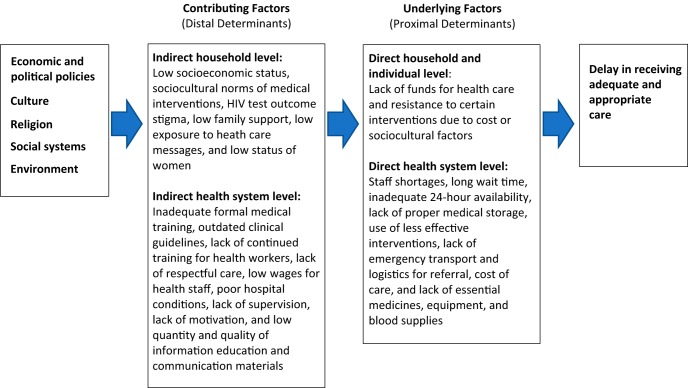
Context of Quality of Health Services for the Third Delay

Reducing deaths related to the third delay requires overcoming barriers to timely, adequate, and appropriate obstetric and neonatal care in facilities.^7^^–^^9^ Many deaths are largely preventable if providers and facilities use the 9 evidence-based medical interventions termed ‘signal functions’ that comprise emergency obstetric and newborn care (EmONC).^10^ Facilities may be classified as providing basic EmONC (BEmONC) if they are able to (1) administer parenteral antibiotics, (2) administer uterotonic drugs for active management of the third stage of labor and prevention and management of postpartum hemorrhage, (3) use parenteral anticonvulsants for the prevention and management of pre-eclampsia/eclampsia, (4) perform manual removal of placenta, (5) perform removal of retained products, (6) perform assisted vaginal delivery, and (7) perform neonatal resuscitation; and classified as providing comprehensive EmONC (CEmONC) if they are able to perform the 7 basic signal functions plus being able to perform a (8) cesarean section and (9) blood transfusion.^6^^,^^10^

### Barriers and Interventions to Improve the Third Delay

Interventions to address the third delay and ensure timely access to the 9 signal functions have concentrated primarily on health facility care during the critical period of labor, delivery, and first 24 hours postpartum when most maternal deaths and about half of newborn deaths occur.^11^^,^^12^ Effective interventions ensure the availability of skilled health providers, sufficient and appropriate medical commodities and equipment, accessible high-quality obstetric care, and high-functioning inter-facility referral and receiving processes.^7^^,^^13^

Human resource shortage is the most frequently cited factor associated with lack of appropriate care in health facilities; it encompasses inadequate training, lack of access to continuing education, staffing shortages, lack of motivation due to poor working conditions and low pay, and lack of optimal supervision and management.^7^^,^^14^ Lack of appropriate medical commodities and equipment is the second most commonly referenced challenge to reducing the third delay, with inadequate drug supply, lack of equipment, and lack of blood cited as common problems.^15^

Human resource shortage is the most frequently cited factor associated with lack of appropriate care in health facilities.

Low demand for facility deliveries and other obstetric services may occur for many reasons, including in low population density areas where there is a need to invest in the availability and accessibility of obstetric care.^16^ Prior negative experiences with unclean, unsafe, or disrespectful care; a lack of competent providers; real or perceived high costs of health care; and cultural beliefs and practices can also contribute to low facility use.^17^^–^^22^ While, the third delay is most directly associated with facility characteristics and quality of care, certain aspects of individual and household barriers, including the negative experiences described above, may contribute to delays in receiving appropriate facility care. Additionally, while delays associated with poor referral practices directly contribute to the second delay, they also contribute to the third delay when facilities delay referral to higher levels or incorrectly refer patients to facilities that cannot provide the level of care needed. Finally, delays in receiving care after arrival at the health care facility contribute to the third delay.^23^^–^^25^ When health facilities have sufficient beds, essential drugs, medical equipment, robust infrastructure, skilled care, and consistent operating hours, women and newborns are more likely to receive appropriate facility care.^26^^,^^27^

### SMGL Context in Relation to the Third Delay

In Uganda, at the beginning of the SMGL initiative in 2011, 57% of all births occurred in a health facility.^28^ Facility births were more common in urban areas (90%) than in rural areas (52%), as were cesarean deliveries (13.7% of urban births, 3.9% of rural births).^28^ The 4 contiguous SMGL districts—Kabarole, Kibaale, Kamwenge, and Kyenjojo—were predominantly rural, with an average population density of 26.2 women of reproductive age per square kilometer and a facility delivery rate of 45.5%, which was slightly lower than the national average of 55%.^28^ At the time the initiative began, national reports found that most health centers in the SMGL Uganda districts had inadequate infrastructure for maternity units, too few functional operating theatres, insufficient numbers of skilled providers, poorly documented health care services and outcomes, and low-functioning referral and communication systems.^29^

In Zambia, 67% of births in 2013 took place in health facilities.^30^ In contrast to Uganda, the Zambia SMGL districts were more sparsely populated, with 3.9 women of reproductive age per square kilometer, requiring longer-distance travel to care.^30^ Cesarean section rates were low in all 3 provinces—3.7% in Eastern Province, 2.9% in Southern Province, and 3.0% in Luapula—reflecting lower rates in rural (3.0%) compared with urban areas (7.2%).^30^ In 2010, 2 of the 3 SMGL districts in Luapula and Eastern provinces were among the 5 provinces with the highest maternal mortality rates and the 3 provinces with the highest child mortality rates.^31^ Poor coverage of maternal and neonatal health services in Zambia was attributed, in part, to weak referral systems, the absence of systems to handle obstetric and neonatal health emergencies, and poor logistics to manage essential drugs.^31^

This article highlights SMGL interventions related to reducing the third delay by ensuring that women and newborns received adequate and appropriate care once at a health care facility. We describe intervention approaches and results in 6 areas in health care facilities necessary to address the third delay:

1. Adequate infrastructure to provide EmONC
2. Sufficient medical supplies, equipment, and medications
3. Sufficient trained health care providers at facilities
4. Improved quality of care and care that is evidence-based
5. Referral capacity to support transfers to higher-level care
6. Effective maternal and perinatal health surveillance

SMGL interventions related to reducing the third delay addressed 6 key areas for improvement: infrastructure, medical supplies and medication, health care provider training, evidence-based care, referrals, and maternal and perinatal surveillance.

Although specific programmatic interventions, detailed in Table 1, varied by location, the overall approaches were aligned.

**TABLE 1.** SMGL Interventions to Reduce the Third Delay, 2011–2016

Strategies and Approaches	Country-Specific Interventions^a^	
	Uganda	Zambia
**Strategy 1. Ensure facilities have adequate infrastructure to provide EmONC**		
Approach 1.1: Support expansion and renovation of operating theaters and facility enhancements to accommodate additional deliveries	Renovated and upgraded operating theaters Increased the size of labor rooms Provided additional delivery beds to allow more women to deliver in facilities and stay longer postpartum	Supported renovation of birthing centers, delivery rooms, and maternity annexes Provided additional delivery beds to allow more women to deliver in facilities and stay longer postpartum
Approach 1.2: Support facility enhancements to improve neonatal survival	Procured incubators, infant warmers, and phototherapy lamps Renovated infrastructure to have designated space for KMC and to create NICUs	Refurbished dedicated KMC rooms at hospitals
Approach 1.3: Support improved access to electricity and water	Provided safe water systems at health facilities Provided solar panels at facilities to improve continuity of access to electricity and light	Improved lighting systems for delivery rooms Improved piped water to maternity annexes
**Strategy 2. Ensure sufficient medical supplies, equipment, and medications**		
Approach 2.1: Strengthen supply chains for essential supplies and medicines	Procured essential medication and backup supply of commodities for all sites on the SMGL project Redistributed supplies between health facilities to reduce stock-outs Implemented SMS reminder system to ensure timely drug ordering Equipped health centers with BEmONC equipment and supplies	Procured essential emergency medications and supplies with backup Trained staff in eLMIS Equipped health centers with BEmONC equipment and supplies Assembled and distributed uterine balloon tamponade kits, and CPAP machines
Approach 2.2: Strengthen availability of blood supplies and surgical equipment	Strengthened and maintained the blood supply system in CEmONC sites and supported new regional blood bank Provided new blood refrigerators Procured and distributed new surgical equipment to facilities	Procured and distributed centrifuges, refrigerators, and freezers to support blood bank Procured and distributed new surgical equipment to facilities
**Strategy 3. Ensure sufficient trained health care providers at facilities**		
Approach 3.1: Recruited staff	Recruited new medical doctors and nurse/midwives through a joint hiring process with the districts	Recruited new nurse/midwives
Approach 3.2: Trained health professionals in emergency obstetric care, including obstetric surgeries	Trained medical officers, anesthetic officers, and midwives/nurses in CEmONC Conducted surgical skills course for medical officers, including decision making and caesarean section Trained providers on neonatal resuscitation/HBB and used drills to reinforce lessons	Trained doctors, nurses, midwives, and anesthetists in EmONC, clinical decision making, obstetric complications, hemorrhage management with uterine balloon tamponade, early HBB, and CPAP Limited rotation of trained providers to different wards Supported capacity building of laboratory staff for blood services
Approach 3.3: Provided mentoring and supportive supervision to newly hired and existing personnel	Conducted individual clinical mentorship sessions Provided selected nurses with intensive hands-on clinical skills placement to expand NICU skills	Trained district mentorship teams who then held monthly on site health facility staff training and mentorship visits on normal delivery and partograph use, EmONC, and HBB
**Strategy 4. Improve quality of care and ensure care is evidence-based**		
Approach 4.1: Implemented quality, effective interventions to prevent and treat obstetric and newborn complications	Provided quality improvement practice to increase partograph use Implemented KMC	Introduced emergency kits and logs/registers to facilitate quick access to emergency supplies Implemented partograph use by facility staff Enhanced infection prevention practices
Approach 4.2: Introduced sound managerial practices using ‘short-loop' data feedback and response to ensure reliable delivery of quality essential and emergency maternal and newborn care	Incorporated concepts related to respectful maternity care into customer care training of midwives Used facility-generated data to review quality of care and implement practice changes	Incorporated respectful maternity care into EmONC and early newborn care and supported it through mentorship
Approach 4.3: Developed guidelines and policies, and ensured protocol adherence	Developed national standards for MDSR that were informed by SMGL processes Implemented BABIES matrix to prevent perinatal deaths by using data to guide actions	Developed clinical guidelines and protocols for diagnosing and managing most common obstetric emergencies Contributed to the development of the newborn health framework and guidelines Created standardized clinical forms to guide providers in recognizing danger signs and diagnosing the most common obstetric emergencies Introduced laminated checklists for quick reference in delivery rooms
**Strategy 5. Ensure referral capacity exists to support transfers to higher-level care**		
Approach 5.1: Improved referral communication systems	Introduced ambulance referral forms to better track referrals Set up and supported district ambulance committees to work on referral-related issues Procured and maintained landline phones for facilities and mobile phones for village health workers	Used referral forms to improve communication between health centers and hospitals Set up and supported district ambulance committees to work on referral-related issues. Repaired and maintained 2-way radios at health facilities. Improved communications through the SMS and Remind-mi mHealth program (local communication programs)
Approach 5.2: Support increased transportation between facilities with motor vehicles or ambulances	Procured ambulances (vehicle and tricycle)	Procured ambulances (vehicle and motorcycle)
**Strategy 6. Support effective maternal and perinatal health surveillance**		
Approach 6.1: Strengthen maternal and perinatal mortality surveillance in facilities and communities	Trained providers on MPDSR Set up MPDSR system, including committees to identify and understand maternal and newborn mortality at facilities and in communities Strengthened prospective health facility surveillance through the MOH DHIS2 Set up POMS and RAPID to understand facility maternal and perinatal mortality Developed national standards for MDSR that were informed by SMGL processes	Established MDSR including verbal autopsies at facilities with a community component Conducted MDR trainings for the district medical officer and health facility staff Supported MDR at facilities
Approach 6.2: Promote a government-owned HMIS data-gathering system to accurately record every birth outcome, obstetric and newborn complication, and treatment at facilities	Trained providers and implemented BABIES matrix Strengthened prospective health facility surveillance through the MOH DHIS2 Set up POMS	Supported national MDSR processes and expansion of MDSR to SMGL districts

a This list is not exhaustive and activities noted may apply to more than one approach.

Abbreviations: BABIES, birth weight and age-at-death boxes for an intervention and evaluation system; BEmONC, basic emergency obstetric and newborn care; CEmONC, comprehensive emergency obstetric and newborn care; CPAP, continuous positive airway pressure; DHIS2, District Health Information System 2; eLMIS, Electronic Logistic Management Information System; EmONC, emergency obstetric and newborn care; HBB, Helping Babies Breathe; HMIS, Health Management Information System; KMC, kangaroo mother care; MDR, maternal death review; MDSR, maternal death surveillance and response; MOH, ministry of health; MPDSR, maternal and perinatal death surveillance and response; NICU, neonatal intensive care unit; NSCU, neonatal special care units; POMS, pregnancy outcome monitoring surveillance; RAPID, Rapid Ascertainment Process for Institutional Deaths; SMGL, Saving Mothers, Giving Life; SMS, short message service.

## METHODS

SMGL used both quantitative and qualitative methods to describe implementation of intervention strategies, outcomes, and health impacts. SMGL implementing partners collected programmatic data throughout the initiative. Partners increased efforts during the initiative to coordinate monitoring and evaluation and harmonize data collection to understand which intervention components were effective. Programmatic interventions detailed here principally occurred in Phase 1 and continued into Phase 2, further details on the content of the phases are described elsewhere.^32^ To evaluate the overall impact of the SMGL initiative, we compared data collected at baseline—the 12 months prior to the onset of the initiative, June 2011 to May 2012—with data collected at the endline, January to December 2016. We collected supplemental qualitative data to describe the influence of specific maternal and perinatal interventions on improving appropriate facility-based care.

### Quantitative Data and Analytic Methods

We used health facility assessments (HFAs), facility-based outcome monitoring, and community-based surveillance to capture key intervention outcomes and health impacts. Approaches and methods for each of these data sources are described in depth.^32^ For our study, we compared maternal and perinatal data collected at baseline and endline.

#### Health Facility Assessments

SMGL partners implemented HFAs in SMGL districts^35^ to assess changes in facility functionality, including facility infrastructure; transportation and communications referral practices; capacity to perform EmONC; equipment and supplies, including essential medicines; staffing, training, and 24-hour availability of medical staff in health facilities; and selected aspects of respectful care. A total of 105 and 110 facilities were assessed at baseline and endline in Uganda and Zambia, respectively. Indicators derived from HFAs that were used in this analysis include basic facility infrastructure and staffing, promotion of protocols and guidelines, availability of essential drugs, performance of EmONC signal functions, and performance of maternal death reviews. Definitions and descriptions of indicators of interest are included in Box 1.

**BOX 1.** Definitions and Descriptions of Indicators of Interest for SMGL Monitoring and Evaluation

Indicator	Description
Performance of EmONC signal functions	Basic Services: Administer parenteral antibiotics Administer uterotonic drugs (e.g., parenteral oxytocin) Administer parenteral anticonvulsants for pre-eclampsia and eclampsia (e.g., magnesium sulfate) Manual removal of placenta Remove retained products (e.g., manual vacuum aspiration, dilation and curettage) Perform assisted vaginal delivery (e.g., vacuum extraction, forceps delivery) Perform basic neonatal resuscitation (e.g., bag and mask) Comprehensive Services: Perform surgery (e.g., caesarean section) Perform blood transfusion
Basic facility infrastructure	Electricity Regular water supply Functional communications systems Motorized vehicles available Services available 24 hours a day
Promotion of protocols and guidelines	Facilities with protocols available and displayed on the following topics: Obstetric and newborn complications Postpartum hemorrhage Active management of the third stage of labor Helping Babies Breathe or kangaroo mother care Early newborn care
Availability of essential drugs	Oxytocin, magnesium sulfate, gentamycin

Abbreviation: EmONC, emergency obstetric and newborn care.

### Facility-Based Outcome Data

Facility-based pregnancy outcome data collection captured clinical data on procedures, complications, and health outcomes. This analysis uses percent of deliveries in EmONC facilities derived from the facility-based data sources.

#### Population-Based Data

We calculated the proportion of deliveries in EmONC facilities with number of live births as the denominator. In Zambia, at baseline, district-specific population from the 2010 national census, external district-specific growth rates, and crude birth rates were used to estimate the number of live births in the SMGL districts.^33^^,^^34^ At endline, the number of live births was determined by applying district-specific facility delivery rates calculated from the 2017 SMGL census.^32^ In Uganda, for both baseline and endline, population statistics were derived from district-wide SMGL household enumerations conducted in 2012 and 2017 in conjunction with the Reproductive Age Mortality Survey studies.^32^

HFAs and pregnancy outcomes monitoring were conducted in virtually all facilities that provide maternity care in SMGL districts. Because we considered data to be complete counts rather than a sample and reported indicators as percentages, our data were not subject to sampling error. We calculated the z-statistic using the McNemar's test for dichotomous responses for matched pairs of data at baseline and endline periods, and calculated relative change in indicators by subtracting the baseline value from endline value and dividing by the baseline value.

### Qualitative Data and Analytic Methods

Qualitative data sources included Phase 1 and Phase 2 project reports, documents submitted by SMGL implementing partners, and special qualitative studies focused on determining the effectiveness of the interventions.

In November 2017, we conducted 16 individual or small group (up to 3 people) in-depth interviews with 28 maternal and newborn health providers from 15 health centers and hospitals in SMGL districts in Uganda. In most cases, the head of maternity identified the most appropriate individuals to participate based on their experience with the Birth Weight and Age-at-Death Boxes for Intervention and Evaluation System (BABIES) matrix. Interviews were conducted to understand the patterns of the matrix's use, its perceived value, and behavior change that resulted from its use. All interviews were conducted in English, audio recorded, and transcribed verbatim, after which common themes were identified from the transcripts.

In November 2017, 16 in-depth interviews were conducted with 28 maternal and newborn health providers from 15 health centers and hospitals in SMGL districts in Uganda.

SMGL introduced BABIES through district workshops, facility-based mentorship, and international conferences. The BABIES report card and storyboards were used to identify ‘trigger’ cells for the BABIES matrix (Box 2). The report card provides a listing of at least 70 indicators that can be calculated from the matrix. SMGL nested the BABIES data within the pregnancy outcome monitoring surveillance database in a multidimensional table to evaluate the third delay using indicators to assess the performance of the system.

BOX 2 **Information and Feedback on the BABIES Matrix**

Saving Mothers, Giving Life (SMGL) used the Birth Weight and Age-at-Death Boxes for Intervention and Evaluation System (BABIES)^35^ matrix as a basic surveillance tool to link maternal and perinatal outcomes with evidenced-based interventions. The BABIES matrix is a simple, expandable, and adaptable table that displays stillbirths and newborn deaths by birthweight and time of death. The matrix accounts for every mother and stillbirth/newborn pair in a facility. Its indicators empower facility staff to monitor and evaluate coverage, equity, and quality-of-care indicators for their patients.

**Figure:**
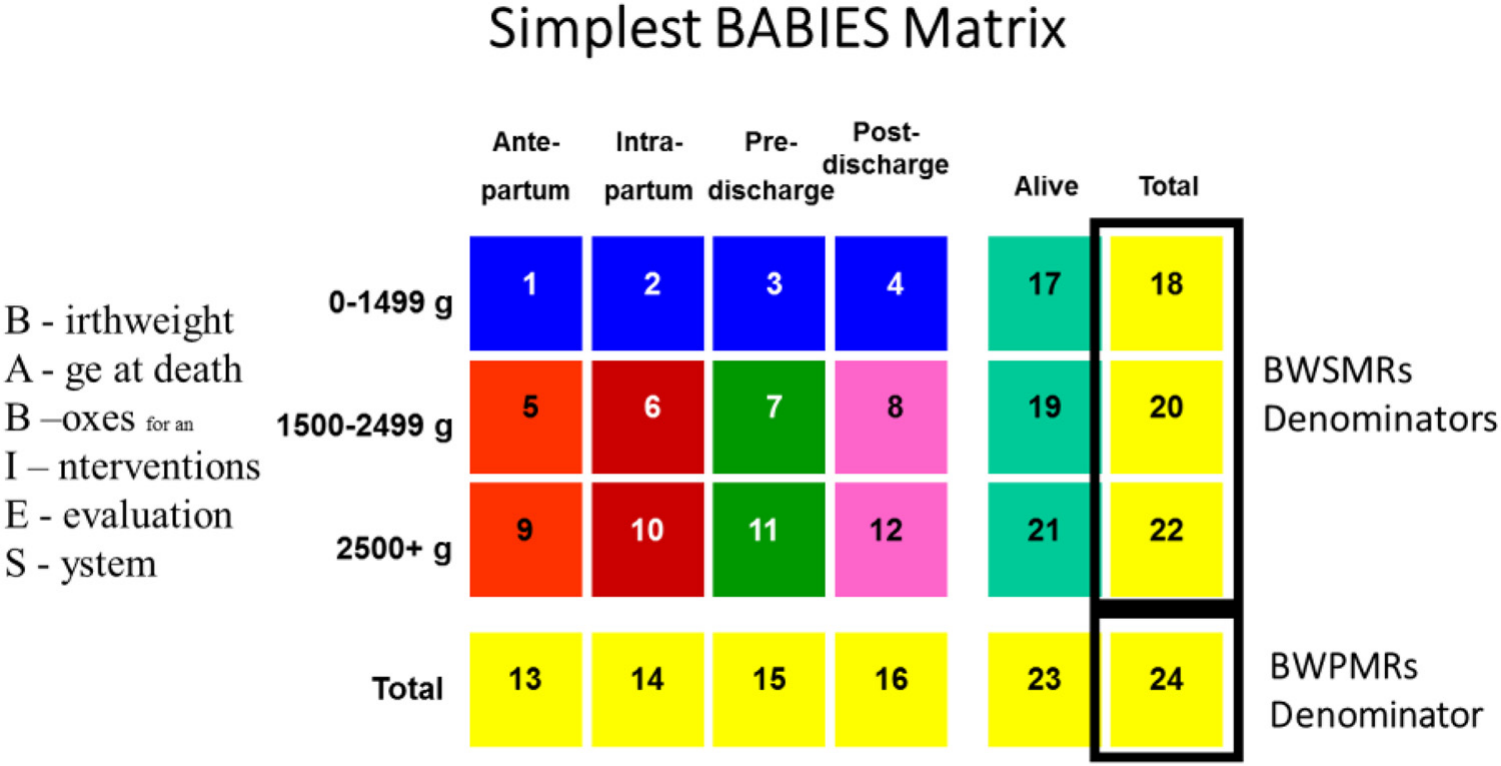

In its simplest form, completion of the BABIES matrix allows facilities to calculate 2 types of mortality indicators. First, birthweight proportionate mortality rates (BWPMRs) help to answer the question “Have we chosen the right thing to do?” Second, birthweight specific mortality rates (BWSMRs) help to answer the question “Are we doing these things right?”

Verbal autopsies collected in the context of baseline and endline population mortality measurement studies captured the causes and circumstances of maternal deaths in both Uganda and Zambia. Questionnaires administered to family members about the women's experiences during the days leading up to a maternal death were used to discern both medical and nonmedical causes of death, such as sociocultural and behavioral factors. Each interview included an open narrative where respondents provided an unprompted account of events preceding the death. Narrative data excerpts were used as a case vignette to illustrate certain barriers to appropriate facility care (Box 3).

BOX 3 **Verbal Autopsy Case Example From SMGL District Verbal Autopsy Case Example of the Third Delay: Sarah's Story**

*Sarah was a 17-year-old from Uganda who died after giving birth to her first child. The respondent during the verbal autopsy interview was Sarah's mother-in-law. The interview was transcribed and summarized below. The names of persons and places and the dates have been changed to protect confidentiality*.

Labor pains started on October 14, 2016 at 8 am. Sarah [the pregnant woman] noted some blood coming from her vagina. She told me what was happening, and I told her to go the hospital if the situation worsened. Instead, Sarah went to work in the garden since the pains were not strong. Early in the morning on the following day, the labor pains increased, and Sarah's husband took us to the hospital on a motorcycle, a 25-minute drive away. Upon reaching the labor ward, Sarah was attended to immediately and soon gave birth. However, shortly after giving birth, she started bleeding heavily, and the midwife started ‘scooping’ blood from the bed and gave Sarah an injection to stop the bleeding. The midwife called the doctor who came immediately. The doctor took Sarah to the operating theatre to establish the cause of the bleeding and told me to go and buy two sachets of blood. I rushed to the health center where I found only one sachet remaining. By the time I purchased the sachet and Sarah was transfused, it was almost 4 pm. I checked on Sarah and found blood coming from her nose; she was unconscious. The nurse told me that they would refer her to a higher level hospital, but I said no, seeing my daughter-in-law in a passing (unconscious) state. Just before 5 pm, right after I completed that statement, Sarah died. The baby remained alive. According to my feeling, the death could not be avoided; it was her time to die—the day that God had planned.

#### Ethics

The study protocol was reviewed and approved by the Ugandan and Zambian Ministries of Health and deemed nonresearch by the U.S. Centers for Disease Control and Prevention Human Research Protection Office of the Center for Global Health. Written informed consent was obtained for respondents in all households and among women for the census, Reproductive Age Mortality Survey study interviews, and verbal autopsies.

## RESULTS

### Strategies, Interventions, and Selected Quantitative Results

The 6 strategies implemented through SMGL are summarized in Table 1 and described below. Outcomes resulting from the implementation of these strategies are presented in Table 2 (Uganda) and Table 3 (Zambia) with selected outcomes highlighted below.

**TABLE 2.** Monitoring and Evaluation Outcomes Associated With Strategies to Reduce the Third Delay in Uganda, 2011–2016 (N=105 facilities)

Indicators	Baseline^a^ Value	Endline^a^ Value	% Relative Change^b^	Sig. Level^c^
**Strategy 1: Ensure facilities have adequate infrastructure to provide EmONC**				
Total number of EMONC facilities	10	25	150.0	N/A
Number of CEmONC facilities	7	17	142.9	N/A
Number of BEmONC facilities	3	8	166.7	N/A
Deliveries in EmONC facilities	28.2%	41.0%	45.4	***
Hospitals/health center IVs that perform blood transfusions^d^	56.3%	100.0%	77.6	N/A
Hospitals/health center IVs that have capacity to perform surgery (caesarean-section)^d^	50.0%	100.0%	100.0	N/A
Facilities with electricity	57.1%	96.2%	68.5	***
Facilities with water	76.2%	100.0%	31.2	N/A
**Strategy 2: Ensure sufficient medical supplies and medications**				
Facilities experiencing no stock-out of oxytocin in the past 12 months	56.2%	81.9%	45.7	***
Facilities experiencing no stock-out of magnesium sulfate in the past 12 months	47.6%	63.8%	34.0	***
Facilities reporting gentamycin antibiotic currently available	90.5%	88.6%	−2.1	NS
**Strategy 3: Ensure sufficient trained health care providers at facilities**				
Facilities reporting at least 1 doctor, nurse, or midwife is on staff	100.0%	100.0%	0.0	NS
Health center IIIs that are open 24/7^e^	74.6%	82.9%	11.1	NS
Facilities reporting EmONC lifesaving interventions performed in the past 3 months^f^				
Parenteral antibiotics	85.7%	92.4%	7.8	NS
Parenteral oxytocin	69.5%	98.1%	41.2	***
Parenteral anticonvulsants	48.6%	34.3%	−29.4	**
Manual removal of placenta	28.6%	54.3%	89.9	***
Remove retained products	19.0%	61.9%	225.8	***
Assisted vaginal delivery	4.8%	10.5%	118.8	NS
Newborn resuscitation	34.3%	87.6%	155.4	***
**Strategy 4: Improve quality of care and ensure care is evidence-based**				
Facilities with protocols and guidelines available and displayed on EmONC lifesaving interventions				
AMTSL	39.0%	58.1%	49.0	***
Postpartum hemorrhage	15.2%	85.7%	463.8	***
Eclampsia or magnesium sulfate use	8.6%	74.3%	764.0	***
Obstetric and newborn complications	26.7%	61.0%	128.5	***
Immediate newborn care	30.5%	79.0%	159.0	***
Facilities that report routine practice of partograph	33.3%	92.4%	177.5	***
Facilities that report routine practice of AMTSL	75.2%	96.2%	27.9	***
Facilities reporting that obstetric patients never share beds	35.2%	91.4%	159.7	***
Facilities reporting that women never deliver on the floor	85.7%	91.4%	6.7	NS
**Strategy 5: Ensure referral capacity to support transfers to higher-level care**				
Facilities with at least 1 method of communication for referrals^g^	93.3%	99.0%	6.1	**
Facilities that reported having available transportation (motor vehicle or motorcycle)^h^	61.0%	59.0%	−3.3	NS
**Strategy 6: Support effective maternal and perinatal health surveillance**				
Facilities with maternal death reviews performed	6.7%	32.4%	383.6	***
Hospital and health center IVs that performed maternal death reviews^d^	31.3%	94.1%	200.6	***

a Baseline period was June 2011 to May 2012; endline period was January to December 2016.

b Percentage change calculations are based on unrounded numbers.

c Asterisks indicate significance levels calculated with a z-statistic using McNemar's as follows: *** = *P*<.01, ** = *P*<.05, NS = not significant. In cases where significance testing is not warranted, this is denoted as N/A.

d Hospital and health center IV was n=16 at baseline and n=17 at endline of HFA.

e Health center III was n=71 at baseline and n=70 at endline of HFA.

f Performance during the previous 3 months preceding the assessment.

g Includes facility owned landline, mobile phone, 2-way radio, or individual had a mobile phone.

h Includes available and functional motorized vehicle with fuel today and funds generally available.

Abbreviations: AMSTL, active management of the third stage of labor; BEmONC, basic emergency obstetric and newborn care; CEmONC, comprehensive emergency obstetric and newborn care; EmONC, emergency obstetric and newborn care; HFA, health facility assessments; N/A, not applicable; NS, not significant; Sig, significance.

**TABLE 3.** Monitoring and Evaluation Outcomes Associated With Strategies to Reduce the Third Delay in Zambia, 2011–2016 (N=110 facilities)

Indicators	Baseline^a^ Value	Endline^a^ Value	%Relative Change^b^	Sig. Level^c^
**Strategy 1: Ensure facilities have adequate infrastructure to provide EmONC**				
Total number of EMONC facilities	7	13	85.7	N/A
Number of CEmONC facilities	4	5	25.0	N/A
Number of BEmONC facilities	3	8	166.7	N/A
Deliveries in EmONC facilities	26.0%	29.1%	12.2	***
Hospitals that perform blood transfusions^d^	100.0%	83.3%	−16.7	N/A
Hospitals that have capacity to perform surgery (caesarean section)^d^	83.3%	83.3%	0.0	NS
Facilities with electricity	55.5%	92.7%	67.0	***
Facilities with water	90.0%	97.3%	8.1	**
**Strategy 2: Ensure sufficient medical supplies and medications**				
Facilities experiencing no stock out of oxytocin in the past 12 months^e^	75.3%	75.0%	−0.4	NS
Facilities experiencing no stock out of magnesium sulfate in the past 12 months^e^	20.0%	43.0%	115.0	***
Facilities reporting gentamycin antibiotic currently available^e^	67.3%	48.2%	−28.4	***
**Strategy 3: Ensure sufficient trained health care providers at facilities**				
Facilities reporting that at least one doctor, nurse, or midwife is on staff	90.0%	98.8%	9.8	**
Health centers that are open 24/7^f^	64.8%	95.5%	47.4	***
Facilities reporting EmONC lifesaving interventions performed in the past 3 months^g^				
Parenteral antibiotics	79.1%	73.6%	−7.0	NS
Parenteral oxytocin	90.9%	95.5%	5.1	NS
Parenteral anticonvulsants	44.6%	40.0%	−10.3	NS
Manual removal of placenta	39.1%	30.0%	−23.3	NS
Remove retained products	17.3%	49.1%	183.8	***
Assisted vaginal delivery	10.0%	15.5%	55.0	NS
Newborn resuscitation	27.3%	74.6%	173.3	***
**Strategy 4: Improve quality of care and ensure care is evidence-based**				
Facilities that report routine practice of AMTSL	71.8%	95.5%	33.0	***
Facilities reporting that obstetric patients never share beds	62.7%	73.6%	17.4	NS
Facilities reporting that women never deliver on the floor	71.3%	83.8%	17.5	NS
**Strategy 5: Ensure referral capacity to support transfers to higher-level care**				
Facilities with at least 1 method of communication for referrals^h^	44.6%	100.0%	124.2	N/A
Facilities that reported having available transportation (motor vehicle or motorcycle)^i^	55.5%	72.7%	31.0	***
**Strategy 6: Support effective maternal and perinatal health surveillance**				
Facilities with maternal death reviews performed	42.5%	75.0%	76.5	**
Hospitals that performed maternal death reviews^d^	50.0%	100.0%	100.0	N/A

a Baseline period was June 2011 to May 2012; endline period was January to December 2016.

b Percentage change calculations are based on unrounded numbers.

c Asterisks indicate significance levels calculated with a z-statistic using McNemar's as follows: *** = *P*<.01, ** = *P*<.05, NS = not significant. In cases where significance testing is not warranted, this is denoted with N/A.

d Hospitals (n=6) included in the HFA.

e Data were not collected in Kalomo facilities so they were excluded from the analysis.

f Health centers (n=88) included in the HFA.

g Performance during the previous 3 months preceding the assessment.

h Includes two-way radio or mobile phone with service.

i Includes motor vehicle, motorcycle, or bicycle.

Abbreviations: AMSTL, active management of the third stage of labor; BEmONC, basic emergency obstetric and newborn care; CEmONC, comprehensive emergency obstetric and newborn care; EmONC, emergency obstetric and newborn care; HFA, health facility assessments; N/A, not applicable; NS, not significant; Sig, significance.

#### Strategy 1. Ensure Facilities Have Adequate Infrastructure to Provide EmONC

Adequate infrastructure is needed to provide safe delivery care and implement EmONC functions. In both countries, SMGL sought to improve basic facility infrastructure and enhance the facilities' ability to provide safe deliveries 24 hours a day/7 days a week (24/7) (Table 1). In Uganda, SMGL supported renovation and upgrading of operating theatres and made facility infrastructure changes to enlarge labor rooms, to create neonatal special care units and to provide spaces for kangaroo mother care, a program that has been shown to reduce neonatal mortality by promoting early skin-to-skin contact and improving thermoregulation in low birth weight and preterm newborns. In Zambia, SMGL increased the number of delivery beds and refurbished rooms to enable increased volume of facility deliveries, longer postpartum stays, and kangaroo mother care. Both countries focused on improving facility availability of electricity and water.

In Uganda, the total number of BEmONC and CEmONC facilities more than doubled between baseline and endline (BEmONC from 3 to 8 and CEmONC from 7 to 17) (Table 2). In Zambia, the number of BEmONC facilities more than doubled (from 3 to 8) and CEmONC facilities increased by 25% (from 4 to 5) (Table 3). This expansion of EmONC facilities is reflected in a corresponding statistically significant increase in the proportion of deliveries occurring in EmONC facilities between baseline and endline for both countries (Uganda from 28.2% to 41.0%; Zambia from 26.0% to 29.1%). The availability of facility electricity increased significantly in both countries (by 69% in Uganda and 67% in Zambia), and water availability in facilities improved (Uganda from 76.2% to 100%; Zambia from 90.0% to 97.3%).

In both Uganda and Zambia, the total number of BEmONC facilities more than doubled between baseline and endline.

#### Strategy 2. Ensure Sufficient Medical Supplies, Equipment, and Medications

In Uganda, SMGL procured essential equipment, including surgical equipment, EmONC supplies, and commodities; strengthened supply chains for essential medicines; upgraded BEmONC facilities; and strengthened the blood supply system in CEmONC sites (Table 1). In Zambia, SMGL supported the procurement of essential medications and implemented logistics management systems to reduce or eliminate stock-outs or supply depletion. Specialized equipment for surgeries, treatment of postpartum hemorrhage, and neonatal resuscitation were obtained for higher-level care along with additional supplies for BEmONC sites. Project C.U.R.E supplied donated facility-specific, essential equipment and commodities shipping 16 containers to Uganda and 20 to Zambia over the life of the initiative.

The proportion of facilities with no stock-outs of oxytocin significantly increased between baseline and endline in Uganda, from 56.2% to 81.9%, but did not change in Zambia. The proportion of facilities with no stock-outs of magnesium sulfate significantly increased in both countries: Uganda increased from 46.7% to 63.8% and Zambia increased from 20.0% to 43.0%. In some cases, however, the availability of essential drugs was unchanged or even declined between baseline and endline. For example, the current availability of gentamicin in Zambia decreased from 67.3% to 48.2%, but remained stable in Uganda at 90.5% at baseline and 88.6% at endline (Table 2 and Table 3).

#### Strategy 3. Ensure Sufficient Trained Health Care Providers at Facilities

In both Uganda and Zambia, SMGL supported recruitment of new providers for facilities in the SMGL districts (Table 1). In Uganda, the facilities hired doctors and nurse midwives at sufficient numbers to meet national standards for newly opened CEmONC facilities, and in Zambia, staffing increases focused on midwives. In Uganda, district leadership elected to maintain midwives and EmONC-trained nurses within the maternity ward rather than rotating staff, which was typical in most facilities. SMGL supported staff training of anesthetists, doctors, midwives, and nurses to expand their skills in the provision of EmONC services (Table 1). In Uganda and Zambia, providers received training in EmONC; clinical decision making; treatment of obstetric complications, including obstetric hemorrhage and eclampsia; surgical skills; and neonatal resuscitation using the Helping Babies Breathe curriculum. In Uganda, clinical officers received surgical skills training on medical decision making and cesarean section. In Zambia, midwives were trained to manage hemorrhages, including using a uterine balloon tamponade to treat postpartum hemorrhage. In both countries, mentorship played a key role in long-term staff development and support, especially for midwives. In Uganda, mentorship included intensive mentoring of obstetricians and individual follow-up visits with mentees.^36^ In Zambia, SMGL implemented a training-of-trainers model where district mentorship teams were trained to mentor others followed by monthly on site training that included drills and clinical mentorship visits.

In both Uganda and Zambia, SMGL supported recruitment of health care staff in order to meet national standards for CEmONC facilities.

In Uganda, the increase in 24/7 care from 74.6% to 82.9% was not significant (Table 2) but, in Zambia, staffing increases contributed to a higher proportion of health centers providing 24/7 care at endline compared with baseline (from 64.8% to 95.5%) (Table 3). Facilities in Uganda reported improvements in the performance of several EmONC signal functions in the 3 months prior to the HFAs, including administration of parenteral oxytocin (from 69.5% to 98.1%), manual removal of placenta (from 28.6% to 54.3%), removal of retained products (from 19.0% to 61.9%), and newborn resuscitation (from 34.3% to 87.6%) (Table 2). There was either a nonsignificant change or reduction in the proportion of facilities performing the remaining signal functions at endline compared with baseline. In Zambia, a significant increase in the proportion of facilities performing 2 signal functions was observed—for removal of retained products (from 17.3% to 49.1%) and newborn resuscitation (from 27.3% to 74.6%)—with no significant change in other signal functions (Table 3).

#### Strategy 4. Improve Quality of Care and Ensure Care is Evidence-Based

Overall, SMGL focused on providing tools to help strengthen provider practices, developing guidelines and policies, and ensuring protocol adherence (Table 1). In Uganda, the project implemented kangaroo mother care in SMGL facilities and trained providers on simplified basic resuscitation, using the Helping Babies Breathe curriculum,^37^ and categorizing infant outcomes by birthweight and age at delivery, using the BABIES clinical tool.^38^ In Zambia, guidelines, protocols, and mini-emergency kits were developed to manage common EmONC emergencies, clinical forms were standardized to guide providers to recognize obstetric danger signs, and checklists were introduced for reference in delivery rooms. Respectful maternity care concepts were introduced into midwife trainings in both countries. Additionally, in both countries, maternal death surveillance and response guidelines were developed and implemented to systematize and strengthen maternal death reviews.

In Zambia, guidelines, protocols, mini-emergency kits, clinical forms, and checklists were introduced or standardized to manage emergencies and improve provider and delivery room safety.

In Uganda, the proportion of facilities with available and displayed protocols and guidelines for lifesaving interventions increased significantly for active management of third stage of labor for prevention of postpartum hemorrhage, management of postpartum hemorrhage, eclampsia treatment, management of obstetric and newborn complications, and immediate newborn care. In Zambia, the proportion of facilities with guidelines displayed was not captured at either baseline or endline.

With regard to the implementation of protocols and practices, in Uganda, the proportion of facilities reporting routine partograph use also increased significantly between baseline and endline (from 33.3% to 92.4%), whereas data were not available in Zambia at both time periods. Partograph use was visually verified by surveyor. The proportion of facilities reporting routine practice of active management of third stage of labor increased significantly in both Uganda (from 75.2% to 96.2%) and Zambia (from 71.8% to 95.5%). The proportion of facilities that reported women never shared beds increased significantly in Uganda (from 35.2% to 91.4%), but not in Zambia (from 62.7% to 73.6%) (Table 2 and Table 3).

#### Strategy 5. Ensure Referral Capacity to Support Transfers to Higher-Level Care

SMGL supported improved referral communication systems and invested in improving transportation between facilities by acquiring additional motor vehicles or ambulances (Table 1). In Uganda and Zambia, referral forms were introduced, ambulances—motor vehicles, motor bikes/tricycles—were procured to assist transportation to and between facilities, and district ambulance committees were formed to strengthen referrals.

In Uganda and Zambia, referral forms were introduced, ambulances were procured to assist in transport to and between facilities, and district ambulance committees were formed to strengthen referrals.

In Uganda, the data showed a significant increase in the proportion of facilities that reported at least 1 method of communication (e.g., telephones and radios) for referral (from 93.3% to 99.0%), but there was no noted improvement in transport availability (from 61.0% to 59.0%) (Table 2). In Zambia, 100% reported having at least 1 method of communication at endline (up from 44.6% at baseline), and the proportion of facilities with available transportation significantly increased (from 55.5% to 72.7%) (Table 3).

#### Strategy 6. Support Effective Maternal and Perinatal Health Surveillance

In both countries, SMGL strengthened maternal and perinatal mortality surveillance in facilities and communities and supported government-managed data processes (Table 1). In Uganda, SMGL helped establish maternal and perinatal death surveillance and response committees, enhanced facility surveillance processes and systems to capture more refined health facility and outcome data, and strengthened the national health management information system. In Zambia, SMGL established the maternal death surveillance and response, including the use of verbal autopsies at facilities and in communities; maternal death review trainings for district and health facility staff; and implementation of death reviews.

The daily visibility of the matrix in the ward was important for increasing awareness and effectiveness.

Significant increases in the proportion of facilities conducting maternal death reviews were observed in Uganda (from 6.7% to 32.4%) and Zambia (from 42.5% to 75.0%). In Uganda, a significantly higher proportion of hospitals and health center IVs reported performing maternal death reviews at endline compared with baseline (from 31.3% to 94.1%). Similarly, in Zambia, 100% of hospitals reported performing maternal death reviews at endline (up from 50.0% at baseline) (Table 2 and Table 3).

### Qualitative Results

The BABIES matrix, implemented in select facilities in Uganda, provided a simple, systematic approach to monitor and evaluate staffing coverage, equity, and quality of care for facility service populations (Box 2).

The aim of the 16 in-depth interviews conducted in November 2017 in Uganda was to better understand the patterns of BABIES matrix use, its perceived value, and behavior change that resulted from its use. Providers described use of the BABIES matrix on a primarily monthly basis during maternity and all-staff meetings and perceived that the matrix was highly valuable, with nearly all providers reporting that it instilled a strong sense of accountability for perinatal deaths that simply had not existed previously. One doctor said, “the first time we ever projected the information, it was an eye-opener. … Oh! It's just not numbers, it's real figures that can influence outcome.” The daily visibility of the matrix in the ward to providers, clients, and visitors alike played an important role in increasing its effectiveness as a tool for awareness.

Providers shared numerous examples of how the BABIES matrix has led to positive changes in the facility, including improved labor monitoring and management, use of the partograph, more honest intercadre communication, better communication and outreach with lower-level facilities, and more complete and accurate documentation, all of which they believe contributed to improved quality of care. When asked if the BABIES matrix will continue to be used after SMGL ends, nearly all providers reported they believed it would. One midwife said, “Yes, whether the SMGL continues, or whether it stops, the BABIES matrix board has to continue. Because whatever we are doing, we are not doing it for SMGL, we are doing it for the better management of our mothers and babies!”

“[W]hether the SMGL continues, or whether it stops, the BABIES matrix board has to continue. Because whatever we are doing, we are not doing it for SMGL, we are doing it for the better management of our mothers and babies!”

Verbal autopsy narratives give context to the facility results by providing descriptions of women's experiences prior to a maternal death. The vignette in Box 3 highlights a woman's experience seeking care and the delays she encountered. This example emphasizes the importance of blood and referrals to facilities that can provide CEmONC.

## DISCUSSION

Successful interventions to reduce maternal and perinatal mortality should ensure that women deliver in facilities with the capabilities and staff to manage both expected and emergent complications. The World Health Organization strongly advocates for all births to be assisted by skilled attendants.^39^ Women are encouraged to give birth in health care facilities to ensure access to skilled health care professionals and timely referral to higher-level facilities for management of obstetric complications, if they occur. However, increasing facility-based delivery rates and EmONC capabilities alone will not ensure that the full range of barriers to appropriate care are addressed. Quality of care depends on a host of factors that SMGL only partially measured. Timeliness and appropriateness of referrals, accuracy of provider decision making and diagnoses, and quality of care provided are only a few factors that contribute to reducing deaths due to the third delay.

### SMGL's Successes

SMGL implemented 6 strategies to reduce deaths due to the third delay by providing the most timely and appropriate delivery care for women and their newborns. In SMGL districts in Uganda and Zambia, the number of CEmONC and BEmONC facilities increased, offering women greater access to maternity services. SMGL enhanced facility capabilities by providing additional infrastructure support and developed processes for medication and supply stocking. SMGL supported increased staffing, trained and mentored staff to implement evidence-based interventions, and provided communication and transportation systems for maternal transfers. SMGL's investments in the availability and accessibility of maternity health services were crucial to increasing the proportion of facility deliveries observed in both Uganda and Zambia and to decreasing maternal and perinatal deaths.^32^^,^^40^

During Phase 2, the SMGL initiative focused on both the mother and newborn. Historically, many EmONC programs have focused heavily on either maternal or newborn health care rather than the provision of effective care for both women and newborns.^41^^–^^43^ Investing in facility-based care for both the mother and newborn, using available interventions, has been found in one study to avert an estimated 71% of newborn deaths, 33% of still-births, and 54% of maternal deaths.^44^ Along with the noted extensive investments in maternal health, SMGL allocated substantial resources dedicated to perinatal survival—improved equipment and expanded training of providers, building of surveillance infrastructure for perinatal mortality, and improved data to action through BABIES. This expanded capacity is likely to have contributed to the overall reduction in perinatal mortality.

SMGL improved data systems, including health outcome information for the monitoring and evaluation of facility performance and accelerated implementation of death reviews to better understand remaining gaps in care and prevent future deaths. The initiative also supported national initiatives and expanded surveillance systems to improve the quality and specificity of maternal and perinatal data for action through maternal death surveillance and response systems. Improved identification, notification, and determination of causes and preventability of maternal deaths allowed decision makers and providers to develop and implement targeted improvements. Expansion of the maternal death surveillance and response system to include perinatal death identification and reviews and implementation of the BABIES matrix in Uganda provided guidance for how to improve newborn health. These enhanced surveillance systems, built on existing data infrastructure and supported by development of national guidelines, are a lasting contribution to national data systems for tracking maternal and perinatal mortality over time.

SMGL improved data systems, including health outcome information for monitoring and evaluating facility performance and accelerated implementation of death reviews to better understand remaining gaps in care and prevent future deaths.

### Limitations of the SMGL Approach and Monitoring and Evaluation Methods

Despite the notable achievements of the SMGL initiative including a documented reduction in facility maternal mortality ratio of 43.8% in Uganda and 37.6% in Zambia, the SMGL endline evaluation found that the maternal mortality ratio in facilities was still unacceptably high: 300 maternal deaths per 100,000 live births in Uganda and 231 maternal deaths per 100,000 live births in Zambia. In both countries, although the majority of women delivered in health facilities (66.8% in Uganda; 90.2% in Zambia), most of the maternal deaths occurred in facilities,^1^ a clear indication that critical gaps remain in improving care and preventing maternal deaths after women reach a health care facility.

The SMGL endline evaluation found that the maternal mortality ratio in facilities was still unacceptably high in both Uganda and Zambia.

More comprehensive studies are needed to document the impact of BABIES on perinatal survival and identify remaining gaps. SMGL efforts demonstrated a reduction in institutional perinatal mortality rates and stillbirth rates in both countries; however, no significant change was found in predischarge neonatal mortality rates. Additional work is also needed to better characterize contributors and interventions needed to impact early neonatal mortality.

SMGL improved facility infrastructure, equipment, and supplies during the initiative. However, the slow and uneven pace of the upgrades was a concern raised during the initiative.^36^ Some sites reported additional needs for refurbishment that were not accomplished, such as further expanding CEmONC capacity, particularly in Zambia.^36^ Additionally, with increased demand for services, the need for essential medications also increased. However, sites noted that the increased supply was not necessarily matched to their need.^36^ Infrastructure and supply chain barriers may have hampered the impact of the initiative on addressing the third delay. The challenges related to these systems are not unique for health programs in resource limited settings, further investment is needed to support these systems in order to achieve real impact.

Although extensive monitoring and evaluation activities were implemented as part of the SMGL initiative, the methods still had important limitations. In Zambia, at baseline, the implementing partners developed unique tools and systems for facility data collection that were not harmonized across districts, and some indicators could not be aggregated at baseline. As census and verbal autopsy data are dependent on secondary reporting by household informants, recall bias may have affected the reported timing and determination of live births, deaths, and health history provided for the deceased. Additionally, the interventions were not evaluated independently, making it impossible to determine the relative impact of any individual intervention.

### Quality of Care is Essential

To address the third delay, identifying barriers in facility-based service provision is critical. In the case vignette of a woman's experience seeking care and the delays she encountered, Sarah's mother-in-law described the facility care and multiple barriers she encountered: lack of active interventions to prevent hemorrhage in the immediate postpartum period, lack of urgent blood transfusion protocol, insufficient blood supply, and a delay in referral to a facility that could provide CEmONC. At each step of her care, these delays compounded Sarah's critical health status. Understandably, stories like these influence other women's decisions about whether to deliver in a facility.^21^

SMGL documented numerous quality-of-care improvements, including increased availability and use of evidence-based protocols and practices. However, SMGL monitoring and evaluation did not capture some dimensions of quality of care, including important areas such as intrapartum monitoring, time from admission or decision to surgery for cesarean deliveries, adequacy of neonatal resuscitation, and effectiveness of care for small and sick babies. Respectful care at birth is an emerging area that SMGL did not measure. Models of quality-of-care assessment that include observational and structured interviews to allow for more refined measurement of maternity care processes and outcomes do exist and would have been a valuable addition to the routine monitoring implemented by SMGL.^45^

Although many quality-of-care improvements were documented, some dimensions of quality of care were not captured.

### Ensuring Sustainability

Additional resources will be required to maintain the advances that the SMGL initiative achieved in addressing barriers that contribute to third delay related maternal deaths. Strengthening and expanding existing national systems for service delivery—including maternal and newborn health, HIV, family planning, and immunization services—are essential to achieve future gains.

The establishment of sufficient human resources for health requires countries to align national and local policies and programs to ensure equitable access to health workers.^46^ In Uganda, the SMGL initiative aligned wages to national standards, transferred human resource management to the national systems to improve sustainability, and limited rotation of specialized staff away from maternities. Expanding preservice training may provide staff with the crucial skills needed sooner, rather than relying on and waiting for in-service training. Tailoring effective interventions to each site would help establish sufficient health care providers for the future. If supported by the national system, task shifting—where tasks are moved to less specialized health workers—can help improve health system efficiency, expand coverage, and save costs.^47^ Other strategies that could be implemented include quality competitions and performance-based financing.^48^^,^^49^

Although SMGL provided extensive health care provider training, the first year evaluation found only a modest increase (10%) in providers' obstetric knowledge.^50^ After the interim assessment, the initiative incorporated active learning, which has been demonstrated to be effective for health workers,^51^ into their skill-building activities; this included interactive obstetric drills and ongoing mentorship, emphasizing the importance of ongoing reinforcement of skills. Further assessment of gaps in knowledge and practice would be useful to tailor trainings to ensure providers maintain and strengthen skills. Linking certification and accreditation with continuing medical education has been implemented in some settings to maintain skills, and models have been developed for low-resource settings.^52^ Investment in ongoing training and sustained mentorship and coaching will help staff retain skills and strengthen clinical practice.^53^ Sites should explore lower-resource intensive models of mentorship to ensure that providers are supported and practices are evidence-based.^54^^,^^55^ Institutionalizing systems for monitoring and retraining staff in national-level policies can help ensure appropriate standards of care. To address that, SMGL enhanced surveillance and strengthened national data collection systems and trained hundreds of facility personnel in improved data capture methods. Further support of these systems is essential to continue to provide improved data for decision making and performance evaluation. Integrating disease surveillance within the national health information system can create a valuable data resource that can inform changes in not only maternal and perinatal mortality but also noncommunicable diseases and emerging epidemics.

Integrating disease surveillance within the national health information system can create a valuable data resource that could inform changes in not only maternal and perinatal mortality but also noncommunicable diseases and emerging epidemics.

## CONCLUSION

The SMGL initiative focused on reducing maternal and perinatal mortality during the critical period around labor, delivery, and immediately postpartum. SMGL implemented 6 comprehensive intervention strategies to focus on the third delay: (1) improving infrastructure to provide EmONC, (2) ensuring sufficient supplies, equipment, and medications, (3) ensuring sufficient trained health care providers at facilities who (4) practice quality evidence-based clinical care, (5) supporting referrals to allow transfers to higher-level care, and (6) supporting effective maternal and perinatal surveillance systems. Implementation of these key strategies was associated with significant reductions in facility maternal and perinatal mortality in Uganda and Zambia over the 5-year SMGL initiative. Further improvements are needed, as maternal and perinatal mortality levels are still unacceptably high. Stakeholders need to leverage the gains made by and sustain the momentum of SMGL and continue efforts to ensure no mother or newborn dies a preventable death.

## Notes

**Peer Reviewed**

**Funding:** Saving Mothers, Giving Life implementation was primarily funded by the Office of the Global AIDS Coordinator, the U.S. Agency for International Development (USAID), Washington, D.C., the Centers for Disease Control and Prevention (CDC), Atlanta, Georgia (Cooperative agreements GPS003057 and GPS002918), Merck for Mothers, and Every Mother Counts. The funding agencies had no influence or control over the content of this article.
